# The magnitude of anemia and preventive practices in mothers with children under five years of age in Dodi Papase, Volta region of Ghana

**DOI:** 10.1371/journal.pone.0272488

**Published:** 2022-08-19

**Authors:** Gideon Dzando, Adekunle Sanyaolu, Chuku Okorie, Urooj Jaferi, Aleksandra Marinkovic, Stephanie Prakash, Risha Patidar, Priyank Desai, Kokab Younis

**Affiliations:** 1 University of Cape Coast, Cape Coast, Ghana; 2 Federal Ministry of Health, Abuja, Nigeria; 3 Union County College, Plainfield, New Jersey, United States of America; 4 All Saints University School of Medicine, Roseau, Dominica; 5 Saint James School of Medicine, Anguilla, BWI; 6 American University of Saint Vincent School of Medicine, Saint Vincent and the Grenadines, Kingstown; 7 Mount Royal University, Calgary, Alberta, Canada; Al-Jouf University College of Pharmacy, SAUDI ARABIA

## Abstract

**Background:**

Socio-demographic factors influence the magnitude of anemia in endemic areas. The purpose of this study is to establish the magnitude of anemia and to determine anemia preventive practices carried out by mothers with children under five years of age in the Kadjebi District of the Volta region of Ghana.

**Methods:**

This purposive sampling study involved women of reproductive age with children less than five years of age from Saint Mary Theresa Hospital and nurses practicing in the pediatric ward of the hospital. A questionnaire and interview guide were administered respectively, and the data collected were analyzed using the Statistical Package for Social Sciences version 21.

**Results:**

A total of 150 participants were studied; among which analysis of anemia was carried out on 129 women whose children had their hemoglobin levels checked and recorded in the laboratory. Hemoglobin levels recorded for these children showed that 85.3% were anemic, even though 93.8% of the mothers had been given iron supplements during their pregnancy. Furthermore, anemia prevention practices comprised whether the child had been given any anti-malaria prophylaxis (98.4% denied), if the child had been dewormed in the last three months (89.9% denied), whether the child was given iron supplements in the last three months (59.7% denied), if the child had been given vitamin supplements in the last three months (24.0% denied).

**Conclusion:**

Most of the children in the study were anemic. Mothers practiced exclusive breastfeeding, iron, and vitamin supplementation, and ensured that children slept under insecticide-treated mosquito nets as a means of preventing malaria.

## Introduction

Anemia is a public health problem that the world is facing and can be defined as a reduced concentration of hemoglobin in the blood [1–3]. This results in a lack of oxygen delivery to vital organs in the body. Anemia in children is a consequence of a wide range of factors, resulting from socio-economic causes such as poor nutrition and poverty; to pathological causes ranging from vitamin deficiencies, and intestinal infections [2, 3]. In other words, nutritional deficiencies, hemoglobinopathies, and infectious diseases are prevalent causes of anemia [3]. Nutritional deficiencies involve the depletion of iron, folate, vitamin B12, and vitamin A [3]. Similarly, infectious diseases like malaria, tuberculosis, HIV, and parasitic infections are considered common causes of anemia [3]. This can lead to negative consequences including increased mortality in children, decreased capacity to learn, impaired physical development, and decreased productivity in all affected individuals. These devastating effects on health, physical, and mental productivity affect the quality of life and translate into significant economic losses for individuals and nations with high anemia prevalence [1, 3]. Anemia is a severe global public health issue that disproportionately affects children under the age of five and pregnant women. According to the 2021 World Health Organization (WHO), 42.0% of children under the age of five and 40.0% of pregnant women are anemic worldwide [3, 4]. Accordingly, laboratory diagnosis of anemia depends on the age of the subject and is defined among those 6–59 months of age with a hemoglobin level greater than 11.0 g/dL to be non-anemic, whereas those less than 7.0 g/dL in the same age range are considered severely anemic [3].

Most African children under the age of five are assumed to be anemic; it is exceedingly frequent even in asymptomatic babies, necessitating the development of preventive interventions to reduce morbidity and death [5]. In Ghana, 78.0% of children under the age of five suffer from anemia, with 23.0% of children being mildly anemic, 48.0% being moderately anemic, and 7.0% being severely anemic [5]. The prevalence of anemia rises with age, peaking at 88.0% for children aged 9 to 11 months and 12 to 17 months, before dropping to 70.0% for children aged 48 to 59 months [5].

Reports from the Ghana national anemia profile showed that the prevalence of anemia in children 6 to 59 months of age declined from 76.1% in 2008 to 65.7% in 2014. The Ghana national anemic profile mentioned numerous probable measures responsible for the decline such as iron and folic supplementation for pregnant adolescents and women of reproductive age, folic acid fortifications, dietary diversity for supplemental feeding, and micronutrient powder for toddlers, as well as a bed net and exclusive breastfeeding [6]. In 2014, 52% of infants in Ghana were exclusively breastfed during the first six months after birth and 59% of children 6–23 months of age consumed foods rich in iron [6].

Mortality related to the high prevalence of anemia in Africa is hard to quantify. This is further complicated by the high prevalence of malaria-associated anemia, which accounts for 190,000 to 974,000 deaths per year in children under five years of age [7].

Documenting the magnitude of anemia in communities in the Kadjebi District may offer fresh insight into potential control strategies. A community-based survey on the magnitude of anemia in children and prevention strategies for anemia among mothers of children under five years of age in the study was conducted at Saint Mary Theresa Hospital.

## Methods

### Study design

A combination of quantitative and qualitative study designs (mixed methods) was used in this study. The quantitative method measured anemia magnitude in children from extracted data on hemoglobin measurement in the hospital laboratory, while the qualitative method was questionnaire-based to assess preventive practices for anemia.

### Variables and concepts

The two variables considered for the study are the magnitude of anemia and anemia preventive practices.

### Setting

The study was conducted at Saint Mary Theresa Hospital, a faith-based hospital situated in Dodi Papase in the Kadjebi District of the Volta region of Ghana.

### Study population

The study includes women of reproductive age 17–49 years and nurses in the hospital. These participants were interviewed within the study period at Saint Mary Theresa Hospital.

### Target population

Mothers with children below five years of age and nurses practicing in the pediatric ward of the hospital.

### Sample and sampling plan

A total of 150 women with children less than five years of age were interviewed using a semi-structured questionnaire. Also, an in-depth interview guide was used to perform interviews with five nurses in the pediatric ward.

### Sampling method

Purposive sampling (criterion sampling for the mothers and convenience sampling for the nurses) method was used for the study. Only mothers with children below five years of age were interviewed at the hospital. Both inpatient and outpatient were interviewed using the questionnaires. Five out of ten nurses in the pediatric unit were also interviewed. Data saturation was reached, and no new information emerged after interviewing the fifth nurse. Furthermore, the socio-demographic characteristics surveyed were the age of the children, sex of the children, marital status of mothers, educational status of mothers, educational status of fathers, parity of mothers, employment status of mothers, employment status of fathers, the religion of mothers, and the number of children under five years of age in the respective families. The socio-demographic characteristics of key nursing respondents included in the study were also examined. These included age, sex, specialty, level of education, years of experience, and years of practice in the children’s ward. Key nursing respondents ranged in age from 24 to 33 years and specialized as registered nurses with a mean overall experience of 4.2 years and 2.2 years of practice in the children’s ward. Of the five nurses, three were of female sex and two were males, and four of the five held a diploma in nursing with one of the five holding a first degree.

### Instruments for data collection

A semi-structured questionnaire with both open and closed-ended questions in the English language was used to collect data from mothers with children under five years of age. The questionnaire which was developed by the authors purposely for the study contained information on demographics, anemia, sickle cell diagnosis in the children, treatment with blood transfusion and iron supplement, and preventive practices. A one-on-one interview of five nursing staff in the pediatric ward was conducted using a structured interview guide to collect data on the subject from a nurse’s point-of-view who tended to the anemic patients.

### Interviewers

Interviews were conducted by three nurses (two males and one female). All Registered Nurses worked in the pediatric and public health units of the hospital and were not part of the responding Nurses in the study. They were recruited and trained strictly for interview purposes.

### Instrument administration procedure

The pre-tested questionnaire was administered to mothers of children less than five years of age directly by cross-questioning them for effective feedback. These respondents were interviewed in a language that they understand. However, in the case where a respondent did not understand English, an expert interpreter for Ewe, or Twi, was readily available to do the translation. With the expert interviewer, a one-on-one interview was held with the nurse respondents using the semi-structured questionnaire. Responses were later transcribed, and a thematic analysis was done.

### Validity and reliability

The interview guide and questionnaire were both vetted and approved by the supervisory team from the School of Nursing and Midwifery, University of Cape Coast, Ghana. They were pre-tested on fifteen mothers with children less than five years of age and three nurses in the children’s ward at the Nkwanta District Hospital and the necessary amendments were made.

### Ethical consideration

Ethical approval for the study was sought from the Institutional Review Board of the University of Cape Coast through the School of Nursing and Midwifery. Approval was also sought from Saint Mary Theresa Hospital, where the study was conducted. An introductory letter from the School of Nursing, University of Cape Coast was submitted to the hospital management of Saint Mary Theresa Hospital for study approval. A consultative discussion was held with the chief, assemblyman, and opinion leaders, to ask for their permission and participation in the study. Consent of the mothers was also sought verbally in the presence of a witness, coupled with the assurance of confidentiality.

### Data analysis

Among the 150 women, an analysis of anemia was carried out on 129 women whose children had their hemoglobin levels checked and recorded in the laboratory. Analysis of anemia could not be carried out on 21 women whose children had a missing record of hemoglobin levels. Data collected were analyzed using the Statistical Package for Social Sciences (SPSS) version 21. Descriptive statistics including frequencies and percentages were used to analyze the research questions of the study and presented on contingency tables. The magnitude of anemia was measured in percentages.

For the qualitative study, the recorded discussions were transcribed verbatim after each interview session. To ensure quality assurance, interview transcripts were compared with notes taken during the interviewing process. Each transcript was further checked by an independent reviewer against the audiotape. Themes were generated and analyzed based on the purpose and objectives of the study.

## Results

### Socio-demographic characteristics

Socio-demographic characteristics survey of respondents’ parents of anemic children are presented in Table 1.

**Table 1:** Sociodemographic characteristics of respondents (parents of anemic children).

Socio-demographic Characteristics	Frequency N = 129	Percent (%)
**Age of Child**		
0–1 month	1	0.8
2–11 months	11	8.5
One-year	26	20.2
Two years	35	27.1
Three years	27	20.9
Four years	29	22.5
**Sex of Child**		
Male	62	48.1
Female	67	51.9
**Marital Status**		
Single	3	2.3
Married	125	96.9
Divorced	1	0.8
**Mother’s Educational Status**		
No education	4	3.1
Basic	2	1.6
Secondary	61	47.3
Tertiary	62	48.1
**Father’s Educational Status**		
No education	1	0.8
Basic	1	0.8
Secondary	17	13.2
Tertiary	110	85.3
**Parity**		
One child	70	54.3
Two children	33	25.6
Three children	12	9.3
Four children	8	6.2
More than 4	6	4.7
**Mother’s Employment Status**		
Unemployed	1	0.8
Farming	7	5.4
Civil servant	60	46.5
Trading	61	47.3
**Father’s Employment Status**		
Farming	6	4.7
Civil servant	96	74.4
Trading	27	20.9
**Mother’s Religion**		
Christianity	89	69.0
Islam	39	30.2
African traditional	1	0.8
**Number of Children < Five Years**		
1	93	72.1
2	32	24.8
3	4	3.1

### The magnitude of anemia in children under five years of age

The magnitude of anemia among children under five years of age was measured using the hemoglobin levels of the children (Table 2). Using the anemia definition, hemoglobin levels less than 11g/dL were considered as being anemic, while those with 11g/dL and above were considered non-anemic. Even among anemic ones, those whose hemoglobin levels are less than 7g/dL are regarded as severely anemic, 7.0g/dL– 9.9g/dL as moderately anemic, and 10.0g/dL– 10.9g/dL as mildly anemic. As shown in Table 2, 44.2% of the children were severely anemic, 32.6% moderately anemic and 8.5% mildly anemic. 14.7% were not anemic. The implication, therefore, is that most of the children surveyed were anemic (85.3%).

**Table 2:** Hemoglobin levels of children.

Hemoglobin Level (g/dL)	Frequency (N = 129)	Percent (%)	Presence of Anemia
Less than 7	57	44.2	Anemic Not anemic
7.0–9.9	42	32.6	
10.0–10.9	11	8.5	
11.0 and above	19	14.7	
Total	129	100.0	

Anemia Prevention by Malaria and Sickle Cell Diagnosis and Treatment with Blood Transfusion and Iron Supplement Anemia preventive practices specific to malaria and sickle-cell diagnosis, as well as treatment with blood transfusion, and iron supplement are shown in Table 3 below.

**Table 3:** Questionnaire response on malaria, sickle cell, blood transfusion, and iron supplement.

Anemia	No		Yes	
N = 129				
	Freq	%	Freq	%
Child given a blood transfusion recently for anemia	76	58.9	53	41.1
Parent being diagnosed with sickle cell	113	87.6	16	12.4
Child being screened for sickle cell disease since birth	111	86.0	18	14.0
Mother being diagnosed with malaria during pregnancy	66	51.2	63	48.8
Mother being given iron supplements during pregnancy	8	6.2	121	93.8

Regarding whether the children received blood transfusion recently as a treatment for anemia, the majority answered “No” (58.9%), while 41.1% answered “Yes.” Many of the respondents also answered “No” to the mothers being diagnosed with sickle cell disease (87.6%); whether the child had been screened for sickle cell disease since birth (86.0%); and whether the mother had been diagnosed with malaria during pregnancy (51.2%). Most of the mothers indicated that they were given iron supplements during pregnancy (93.8%).

### Anemia preventive practices

Anemia prevention practices examined in Table 4, comprised whether the houses had been sprayed with insecticide in the last twelve months (frequency of 88 respondents at 68.2% did not, whereas 41 at 31.8% did), whether the child slept under a treated mosquito bed net the previous night (frequency of 69 respondents at 53.5% did not, whereas 60 at 46.5% did), if the child had been given any anti-malaria prophylaxis (frequency of 127 respondents at 98.4% did not, whereas 2 at 1.6% did), whether the child had been dewormed in the last three months (frequency of 116 respondents at 89.9% did not, whereas 13 at 10.1% did), whether the child was given iron supplements in the last three months (frequency of 77 respondents at 59.7% did not, whereas 52 at 40.3% did), whether the child had been given vitamin supplements in the last three months (frequency of 31 respondents at 24.0% did not, whereas 98 at 76.0% did), and whether the child was exclusively breastfed (frequency of 49 respondents at 38.0% did not, whereas 80 at 62.0% did).

**Table 4:** Anemia preventive practices.

Practices	No		Yes	
N = 129				
	Freq	%	Freq	%
House being sprayed in the last 12 months	88	68.2	41	31.8
Child sleeping under treated mosquito net previous night	69	53.5	60	46.5
Child ever been given malaria prophylaxis	127	98.4	2	1.6
Child being dewormed in the last three months	116	89.9	13	10.1
Child being given iron supplement in the last three months	77	59.7	52	40.3
Child being given vitamin supplements in the last three months	31	24.0	98	76.0
Child being exclusively breastfed	49	38.0	80	62.0

Knowledge of mothers on anemia preventive strategies was examined. The knowledge of mothers on anemia preventive strategies showed that half of the respondents indicated that eating good food and averting malaria help to prevent anemia (50.0%). While 40.7% said eating good food alone helps to prevent anemia, 4.7% of the respondents said good food and clean drinking water prevent anemia, and 4.7% indicated that good food and sleeping well help to prevent anemia.

## Discussion

### Socio-demographic characteristics

Socio-demographic characteristics of women surveyed comprised age of the children, sex of the children, marital status of mothers, educational status of mothers, educational status of fathers, parity of mothers, employment status of mothers, employment status of fathers, the religion of mothers, and the number of children under five years of age.

With regards to the age of children, infants constituted 0.8%, while those four years old formed 22.5%. Mothers with children two years of age, however, formed the comparative majority (27.1%) while mothers with children three years of age constituted 20.9%.

Mothers with children, who were female, were in majority (51.9%), while males constituted 48.1%. Most of the women indicated that they were married (96.9%), while 2.3% said they were single. With regards to the level of education, while 48.1% of mothers said they had tertiary education, 85.3% also indicated that the fathers of the children had tertiary education. About 3.1% and 0.8% of mothers and fathers respectively, had no education. Most of the mothers had only one child (54.3%), while 4.7% gave birth to more than four children. Out of this, 72.1% said they had one child under the age of five years, while 3.1% had three children under five years of age.

While most of the mothers were traders (47.3%), the fathers of children under five were civil servants (74.4%). Only 5.4% and 4.7% of mothers and fathers respectively, were engaged in farming. Christianity was the most dominant religion among the respondents (69.0%), while Islam had a 30.2% representation.

Findings of this study concerning sex and marital status, are consistent with findings of the 2010 population and housing census, which places females as the majority with 51.2%, being married as the most prevalent marital status among adults eighteen years of age and above. The census also indicated that Christians form most of the Ghanaian population in terms of religion, followed by Islam [8].

### The magnitude of anemia in children under five years of age

Many of the children were severely anemic, moderately anemic, or mildly anemic. This was measured by using the available hemoglobin level estimation values of the children, which showed that most of them were below 11g/dL. The implication, therefore, is that anemia is very prevalent among children under five years of age. As identified in this current study, severe anemia is more prevalent than mild and moderate anemia at Saint Mary Theresa Hospital in Dodi Papase regardless of the causative agent. These results corroborate findings from key informants which also indicate that there is a high magnitude of anemia among children in the district. The elevated levels of anemia among children were corroborated by interviews conducted with the key informants. These five interviewees all confirmed that anemia is very prevalent at the hospital. One respondent, for instance, had this to say:

‘Anemia is one of the most common conditions among children [at this facility]. There are times when we have only children with severe anemia admitted to the ward and there are no spaces for mild and moderate anemia admissions. Here, anemia cases are just serious’ (Respondent 3, male, 33 years).

Findings of this study with 73.3% of the children having anemia corroborate with the study of Ewusie *et al*. 2014 which reported a prevalence of 78.4% for anemia in under-five children in Ghana [5].

### Anemia preventive practices specific to malaria and sickle cell diagnosis, as well as treatment with blood transfusion, and iron supplement

Further preventive practice based on diagnosis and treatment, realized by the study, are parents of children being diagnosed with sickle cell, the child not being screened for sickle cell disease since birth, the mother being diagnosed with malaria during pregnancy, the child not being given iron supplements in the last three months, worm infestation, and kidney disease. Although blood transfusion is a treatment for anemia, 41.1% of the mothers indicated that their children received blood transfusion recently for anemia.

While iron deficiency is frequently the main factor that contributes to anemia, it is of importance to recognize that a multisectoral approach through integrated interventions that address the numerous factors that play a significant role in producing anemia in each community is required to control it [9]. Iron deficiency, infectious diseases such as malaria, helminth infections, and other chronic infections, particularly HIV/AIDS and tuberculosis, as well as other nutritional deficiencies, are important associated risks of anemia in children [3, 9–11].

Fifty percent of all associated risks of anemia worldwide are due to inadequate dietary intake of bioavailable iron, increased iron requirements during rapid growth periods (such as pregnancy and infancy), and increased blood loss due to hookworm or schistosome infestation [12, 13]. The most common associated risks of anemia in Ghana are inadequate dietary intake of iron, malaria infection, and intestinal worm infestation [14].

Response from the key informants indicated that worm infestation and kidney disease, are responsible for anemia among children. One respondent, for instance, said:

‘Most mothers do not take good care of their children by giving them a less nutritious diet. Mothers also leave their children to play without any protection against mosquito bites. Malaria is therefore mainly responsible for their anemic statuses. Kidney diseases and sickle cell diseases also cause it [anemia]’ (Respondent 1, female, 27 years).

### Anemia preventive measures

The study identified a few anemia preventive techniques, including home spraying and children sleeping under treated mosquito nets. Furthermore, those who received malaria prophylaxis and those who were dewormed had more preventative measures. Additionally, children who received iron and vitamin supplements, as well as those who were exclusively breastfed, had better outcomes.

Concerning the findings of this study, Ewusi *et al*. 2014 [5] noted that much emphasis needs to be placed on increasing awareness of anemia in the community by healthcare workers and significant others. Mild and moderate anemia in children under five years of age, if unrecognized, may lead to the severest form with unbearable complications that may be life-threatening.

Ewusi *et al*. 2014 [5] argued that although blood transfusion [1] and other treatment modalities are available for treating anemia, the single most definitive and surest way to prevent the burden of anemia on families and nations is to adopt preventive measures to curb the occurrence of anemia. According to Atinmo *et al*. 2009 [12], supplementing dietary iron with iron tablets, syrups, drops, or elixirs, and fortifying processed foods and condiments with iron is the best defense against iron deficiency anemia. Similarly, a study by Uyoga *et al*. 2020 on Kenyan infants, reported that morning and afternoon doses of 12 mg oral iron are equally absorbed in newborns and that dosing on consecutive days increases plasma hepcidin while slightly decreasing iron absorption compared to alternate-day dosing [15]. In addition, dosing on alternate days or every third day did not affect plasma hepcidin or absorption of iron; because high plasma hepcidin decreases iron absorption, alternate day iron dosing is recommended [15]. Where fortification has been evaluated in specific populations, it has improved iron status and reduced anemia prevalence [12]. Also, approximately 60.0% of anemia in infancy could be prevented by anti-malarial chemoprophylaxis [2, 16]. The formulation of preventative efforts should center on caregiver impressions of what steps may be made to prevent or cure anemia.

In the qualitative study, the key informants corroborated the strategies indicated in the mothers’ questionnaire as the means of preventing anemia among children under-five years of age. In addition to these, the informants indicated that mothers should always feed their children a good diet. Two of them had these to say:

a. Mothers should feed their children a good diet. We have a lot of vegetables here, yet they don’t know that vegetables even boost the child’s hemoglobin. Mothers should also do well to protect their children from malaria. Mothers should feed their children a good diet… blood transfusion seems the only surest way of ensuring that the decreased blood level is increased. So, children with severe anemia are mostly transfused (Respondent 2, male, 24 years).

b. Mothers should give their children good food, especially foods rich in iron which are inexpensive but not identified by the mothers. For example, green leafy vegetables. Also, mothers should closely monitor their children to detect early changes in their health to avoid complications (Respondent 4, female, 28 years).

### Limitations to the study

Limited-time period and external factors including lack of finances, large sample size, unavailability of respondents, and language barriers were limitations to the study. Also, the number of confounding factors and pertinent clinical variables considered in this study is limited. The study was performed over one month in the early rainy season, and hence seasonal variations in the pattern of anemia cannot be discerned. Furthermore, 129 out of 150 mothers who participated in this study had their children’s hemoglobin levels checked in the laboratory due to presenting clinical manifestations. Women who were excluded may have caused data bias and skewed the result. In addition, other limitations include the inability to run comparative statistical analyses and the calculation of the power retrospectively.

## Conclusion

Nutritional deficiencies, worm infestation, and malaria were identified as the major associated risks of anemia among the study children. Mothers adopted measures such as allowing their children to sleep under insecticide-treated nets, exclusively breastfeeding them, and giving the children iron and vitamin supplements to prevent anemia. Recommendations to prevent anemia in children below five years of age in a developing country like Ghana, include checking hemoglobin levels regularly, providing malarial prophylaxis, educating hospitals and staff about how to detect anemia early, and organizing blood donation programs to keep up with blood supply in the hospital as well as educating the mothers about the plausible causes and prevention of anemia. There is a need for further research to be conducted especially in subgroups of the population with a high prevalence to determine potential risk factors associated with the magnitude of childhood anemia. Further studies are also needed to determine appropriately targeted interventions as the prevalence of anemia in rural communities in Ghana is deemed high, as well as research on the outcome of treated anemia cases for follow-up.

## Supporting information

S1 File Questionnaire and interview guide. (DOCX)
