# Supplementary material for: The magnitude of anemia and preventive practices in mothers with children under five years of age in Dodi Papase, Volta region of Ghana
Source: PLoS One. 2022 Aug 19;17(8):e0272488. doi: 10.1371/journal.pone.0272488 (PMC9390896; doi:10.1371/journal.pone.0272488)
Supplement: S1 File — (DOCX) [file pone.0272488.s001.docx]

**APPENDIX**

**UNIVERSITY OF CAPE COAST**

**COLLEGE OF HEALTH AND ALLIED SCIENCES**

**SCHOOL OF NURSING AND MIDWIFERY**

Hello, my name is Dzando Gideon. I am a Nursing student of the University of Cape Coast and conducting a research on **The Prevalence, Associated Risks, and Anaemia Preventive Practices Among Mothers With Children Under Five Years of Age.** The data collection forms part of my academic work. I would be glad if you could take time off your busy schedule to respond to this questionnaire for me. Your responses will be confidentially kept and will not be exposed to unauthorised access. Thank you for accepting to be a part of this study.

**Consent**: I have given my consent to participate in this study YES { } NO {}

**Instructions:** Please tick or write where appropriate

**Section A: Socio-demographic characteristics**

- 1. How old are you? (Mothers age) Please write...............
  2. Location Please write................
  3. How old is your child? Please write...............
  4. What is the sex of your child Male { } Female { }
  5. Marital status Single { } Married { } Divorced { } Widowed { } co-habitation { }
  6. Mother’s educational level No education { } Basic { } Secondary { } Tertiary { }
  7. Father’s educational level No education { } Basic { } Secondary { } Tertiary { }
  8. Parity of Mother please write...................
  9. Mother’s employment status Unemployed { } Farming { }

Civil servant { } trading { } Other (specify)..................

- 1. Father’s employment status Unemployed { } Farming { } Civil servant { } trading { } Other (specify).................
  2. Mothers Religion Christian { } Muslim { } Traditionalist { } Other (specify).............
  3. How many of your children are below age five (5)? ..................................

**Section B: Prevalence of Anaemia in Children Under-Five**

- 1. Haemoglobin level estimation (if any) in the past 3 months Please write.................
  2. Has your child ever been diagnosed with Anaemia?

Yes { } No { }

- 1. Has your child been diagnosed with Anemia in the last 6 months?

Yes { } No { }

**Section C: Associated Risk of anaemia among children under five**

- 1. Has your child received any blood transfusion recently? YES { } NO { }
  2. Have you (parents) been diagnosed with sickle cell disease (SCD) in the past?

YES { } NO { }

- 1. Has the child been screened for SCD since birth? YES { } NO { }
  2. Were you diagnosed with malaria during pregnancy?
  3. During pregnancy, were you given iron supplements? YES { } NO { }
  4. Do you have any food totems? YES { } NO { }
  5. If yes, what are they? Please write

..............................................................................................................................................................................................................................................

**Section D: Anaemia preventive practices**

- 1. Have you sprayed your house with indoor residual spray in the past 12 months? YES { } NO { }
  2. Did your child sleep under insecticide treated mosquito net last night?

YES { } NO { }

- 1. Have your child experienced fever in the past Two weeks?

YES { } NO { }

- 1. If yes, what did you do? Please write ..............................................................................................................................................................................................................................................
  2. Have you ever given Malaria prophylaxis to your child?

YES { } NO { }

- 1. Have you de-wormed your child in the last three months?

YES { } NO { }

- 1. Have you given your child any iron supplement within the last 3 months?

YES { } NO { }

- 1. Have you given your child any vitamin supplement in the past 3 months?

YES { } NO { }

- 1. Did you exclusively breastfeed your baby? YES { } NO { }
  2. If yes, How many months? Please write......................................
  3. How many times do you feed your child in a day? Please write.............
  4. What are the sources of iron in your diets?

..............................................................................................................................................................................................................................................

- 1. How do you think anemia among children can be prevented?

Please write

..............................................................................................................................................................................................................................................

UNIVERSITY OF CAPE COAST

SCHOOL OF NURSING AND MIDWIFERY

**INTERVIEW GUIDE FOR KEY INFORMANTS**

Hello, my name is Dzando Gideon. I am a Nursing student of the University of Cape Coast and conducting a research on **The Prevalence, Associated Risks and** **Anaemia Preventive Practices Among Mothers With Children Under Five Years of Age.** The data collection forms part of my academic work. I would be glad if you could take time off your busy schedule to respond to this questionnaire for me. Your responses will be confidentially kept and will not be exposed to unauthorised access. Thank you for accepting to be a part of this study.

**Section A: Socio-demographic Characteristics**

1. Age:
2. Sex:
3. Nursing specialty:
4. Rank:
5. Level of Education:
6. Years of Experience:
7. Years of practice in the children’s ward:

**Section B: Prevalence of Anaemia in Children Under-five**

1. In your practice of Nursing in the children’s ward, how will you describe the number of anaemia cases that report in the hospital?
2. How will you describe the number of anaemia cases that report back to the facility post discharge with same anaemia?

**Section C: Associated risks of anaemia among children under five**

1. What are the causes of anaemia in children below five years?
2. What role do mothers play aiding the anaemia in children below five years of age
3. What other diseases, if any, can result in Anaemia?

**Section D: Anaemia preventive practices**

1. What are some challenges in preventing anaemia in the houses as speculated by mothers?
2. What are some complications that might evolve due to untreated anaemia?
3. What role can mothers play in your view in preventing anaemia in children below five years?
4. How does blood transfusion help in preventing anaemia in children?

Thank you for your Cooperation.
